# The Method Used in Germany in the Treatment of Placenta Prævia

**Published:** 1879-12

**Authors:** Louis Schade


### Translations.

---

### THE METHOD USED IN GERMANY IN THE TREATMENT OF PLACENTA PRÆVIA.

ABSTRACT OF PROCEEDINGS OF THE BERLIN GYNECOLOGICAL ASSOCIATION (GESELLSCHAFT FUER GEBURTSHUELFE) OF JANUARY 23, 1877.

TRANSLATED BY LOUIS SCHADE, M. D.

Professor Schraeder says: As regards the occurrence of bleeding in connection with placenta prævia, the views of Duncan that the placenta detached itself in consequence of transverse tension of the internal os and the parts adjacent (by which tension the attachment became larger while the placenta retained its original size), did not seem to him to explain every case, nor did it necessarily explain the hemorrhage. In normal births, where the membranes have ruptured at the proper moment, the ovum must partially detach itself from the wall, and this last must draw itself back over the ovum. This separation takes place usually in decidua, sometimes, however, between chorion and amnion. In this case the chorion is torn, remains attached to the wall of the uterus, and the amnion alone constitutes the membrane. After delivery of the fœtus one finds both separate. If now, by placenta prævia centralis, the ovum remains intact, the internal os must force itself up on the placenta, and in consequence of this, the projecting portion becomes apparently larger. A separation between amnion and the chorion is almost impossible on account of the position of the umbilical cord. As soon, however, as the membranes break, the wall of the uterus ceases to draw itself up on the placenta. From this it appears that the earliest possible rupture of the membrane must be the safest check to the bleeding, and indeed this treatment has had the best results. It is a mistake to suppose that the bleeding can be stopped by compression of the presenting portion. In a few of the cases treated in the lying-in hospital, the bleeding ceased immediately on rupturing the membrane, although only a thigh lay in the orificium uteri, which did not at all fill it out. A forced birth is, however, not advisable, as the cervix in placenta prævia, although it may be dilated, is also easily ruptured.

Dr. Bennicke has, in nine out of twelve cases, treated in the city, ruptured the membrane early; then by a combined version pulled down a foot, and then allowed nature to finish the delivery. The twelve women lived, also four of the children.

Dr. A. Martin ended forty-one cases operatively, and lately has advocated as the safest the treatment herein prescribed. He recommended the rupture of the membranes because the disadvantage of the tampon, especially the infection, is thereby avoided. The cervix is not always easily dilated; for instance, he once saw a case of rupture occurring during the passage of the head, and by which the woman bled to death. He tried, only once, Kleinwaechter's method to loosen the placenta considerably, and then to inject ice water, and with bad result.

After the birth of the child the placenta must be immediately removed. Against post-partum hemorrhage he recommends injection of hot water in the uterus.

Dr. Jacquet expresses himself also in favor of early rupture of the membrane and of combined version.

